# High-Performance Flexible Energy Storage Devices Based on Graphene Decorated with Flower-Shaped MoS_2_ Heterostructures

**DOI:** 10.3390/mi14020297

**Published:** 2023-01-23

**Authors:** Yongteng Qian, Zhiyi Lyu, Qianwen Zhang, Tae Hyeong Lee, Tae Kyu Kang, Minkyun Sohn, Lin Shen, Dong Hwan Kim, Dae Joon Kang

**Affiliations:** 1 College of Pharmacy, Jinhua Polytechnic, Jinhua 321007, China; 2 Department of Physics, Sungkyunkwan University, 2066, Seobu-ro, Jangan-gu, Suwon-si 16419, Republic of Korea; 3 School of Chemical Engineering, Sungkyunkwan University, 2066, Seobu-ro, Jangan-gu, Suwon-si 16419, Republic of Korea

**Keywords:** graphene, MoS_2_, heterostructures, flexible supercapacitor, energy storage devices

## Abstract

MoS_2_, owing to its advantages of having a sheet-like structure, high electrical conductivity, and benign environmental nature, has emerged as a candidate of choice for electrodes of next-generation supercapacitors. Its widespread use is offset, however, by its low energy density and poor durability. In this study, to overcome these limitations, flower-shaped MoS_2_/graphene heterostructures have been deployed as electrode materials on flexible substrates. Three-electrode measurements yielded an exceptional capacitance of 853 F g^−1^ at 1.0 A g^−1^, while device measurements on an asymmetric supercapacitor yielded 208 F g^−1^ at 0.5 A g^−1^ and long-term cyclic durability. Nearly 86.5% of the electrochemical capacitance was retained after 10,000 cycles at 0.5 A g^−1^. Moreover, a remarkable energy density of 65 Wh kg^−1^ at a power density of 0.33 kW kg^−1^ was obtained. Our MoS_2_/Gr heterostructure composites have great potential for the development of advanced energy storage devices.

## 1. Introduction

Due to their exceptional chemical and physical properties, transition metal dichalcogenides (TMDCs) have been widely employed as active materials in supercapacitors (SCs) [1,2,3]. Of the numerous TMDCs that have been synthesized, MoS_2_ is a remarkable electrode material for use in SCs as it has a unique structure, high electrical conductivity, and is eco-friendly [4,5]. However, the electrochemical performance of MoS_2_ is significantly limited by its low energy density and poor long-term cycling stability. To address these issues, extensive efforts have been directed toward improving the electrochemical performance of MoS_2_ via a range of different strategies, including modification of MoS_2_‘s structure [6,7], doping with non-metal ions [8,9], hybridization with metal oxides [10,11], and integrating it with highly conductive materials [12,13,14]. Despite significant advances in the synthesis of MoS_2_, the overall electrochemical behavior of MoS_2_-based SCs is still far from satisfactory.

Recently, graphene (Gr), an archetypal two-dimensional (2D) material, has attracted a considerable amount of attention due to its superior properties, such as its high stability and large specific surface area, as well as high thermal and electrical conductivities [15,16,17]. Due to these excellent properties, Gr has been explored in many research fields, including nanogenerators, SCs, field-effect transistors, photodetectors, etc. [18,19,20,21]. In particular, utilizing Gr as a co-active material in combination with MoS_2_ to construct MoS_2_/Gr composites has proven to be an efficient strategy in significantly improving the electrochemical properties of MoS_2_, such as its electrical conductivity, structural stability, and surface area, as well as its carrier charge-transfer rate [22,23,24,25,26]. For instance, Saraf et al. prepared MoS_2_/rGO hybrids as an electrode material for SCs. These MoS_2_/rGO hybrid-based SCs exhibited an excellent electrochemical capacitance of 387.6 F g^−1^ at 1 A g^−1^ [23]. Jia et al. fabricated MoS_2_/Gr composites as active materials for SCs, which showed a remarkable electrochemical capacitance of 445.7 F g^−1^ at 0.8 A g^−1^ [25]. However, the synthesis of high-performance MoS_2_/Gr composites for SCs is still limited by their low energy density as well as their poor long-term cycling stability. Motivated by these challenges, a unique heterostructure was rationally designed by integrating flower-shaped MoS_2_ with Gr to take advantage of the following benefits: (i) the Gr layer enhances the electrical conductivity of the electrode; (ii) the large surface area of the Gr layer provides abundant growth sites for the subsequent growth of MoS_2_; (iii) flower-shaped MoS_2_ provides ample ion transfer channels and abundant surface active sites in the electrochemical reaction process; and (iv) the MoS_2_/Gr heterostructure increases the interfacial contact area, enhances the structural stability of compounds, and facilitates ion transfer between Gr and MoS_2_, thus improving its electrochemical performance.

In this study, a low-cost strategy is reported for fabricating flexible, high-performance MoS_2_/Gr heterostructure-based SCs. In a three-electrode system, the MoS_2_/Gr heterostructure exhibits a high specific capacitance, i.e., 853 F g^−1^ at 1.0 A g^−1^. Furthermore, a flexible asymmetric SC (ASC) device was constructed, comprising the MoS_2_/Gr heterostructure and activated carbon as positive and negative electrodes, respectively. The flexible ASC device exhibited an exceptionally high electrochemical capacitance of 208 F g^−1^ at 0.5 A g^−1^, as well as a superior energy density of 65 Wh kg^−1^ at a power density of 0.33 kW kg^−1^. It is worth noting that 86.5% of the electrochemical capacitance of the flexible ASC device was retained after 10,000 cycles at 0.5 A g^−1^, indicating its long-term cycling durability. The superior electrochemical properties of the MoS_2_/Gr heterostructure electrode can be ascribed to MoS_2_ having abundant surface reaction active sites, with Gr boosting the electrical conductivity of the electrode, in addition to the aforementioned strong synergistic effects of the MoS_2_ and Gr. Thus, in this approach, the drawbacks reported in the literature can be overcome, with the flexible ASC device exhibiting a high energy density, long-term cycling durability, and high flexibility. Our results indicate that the MoS_2_/Gr heterostructure is thus an excellent active material for use in sustainable advanced SCs.

## 2. Materials and Methods

### 2.1. Fabrication of MoS_2_/Gr Heterostructures

**Scheme 1:**
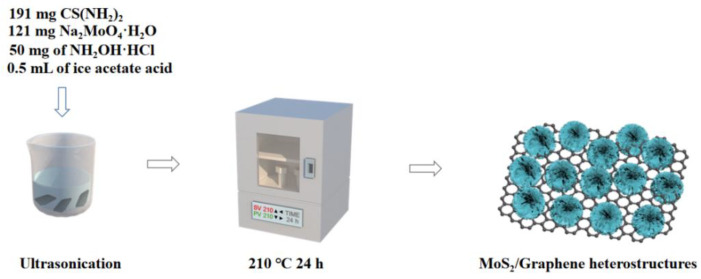
Preparation procedure of the MoS_2_/Gr heterostructure.

The Gr oxide was fabricated via the modified Hummers approach [27]. The MoS_2_/Gr heterostructures samples were synthesized via a facile hydrothermal method (Scheme 1). First, 50 mg of Gr powder was dispersed in 15 mL of distilled water through ultrasonication. Second, 121 mg of Na_2_MoO_4_·H_2_O and 191 mg of CS(NH_2_)_2_ were added to the above suspension, and ultrasonication was performed for 40 min. Third, 50 mg of NH_2_OH·HCl and 0.5 mL of ice acetate acid were added to the above suspension solution, while continuous ultrasonication was performed for 20 min. The mixed solution was then transferred to a Teflon autoclave and kept at 210 °C for 24 h. It should be noted that, after the hydrothermal reaction process, the Gr oxide was reduced to Gr under NH_2_OH·HCl as a reducing agent. Subsequently, the samples were collected via centrifugation and rinsed three times using distilled water and ethanol, separately. The product was then dried at 70 °C for 10 h in a vacuum oven to obtain MoS_2_/Gr heterostructures. The product characterizations are displayed in the Supporting Information.

### 2.2. Materials’ Characterizations

The structures and crystalline phases of the as-prepared powder were examined using X-ray diffraction (XRD, 40 kV × 40 mA, Rigaku Ultima III, Tokio, Japan) with Cu-K_α_ radiation (λ = 1.5418 Å). The measured 2θ range was from 10° to 70° with a scanning speed of 5°/min. The XRD result was corrected to an apparatus line width of 0.08. The chemical composition of the samples was analyzed via X-ray photoelectron spectroscopy (XPS, Thermo Scientific, Nottingham, UK). The XPS was carried out with a standard Al-Kα source (1486.6 eV) in an ultra-high vacuum (~8 × 10^−9^ Pa). The binding energies were corrected for the charging effects by considering the adventitious C 1s line at 284.6 eV. The morphology of the product was examined by scanning electron microscopy (SEM, JEOL JSM-7400E, Tokyo, Japan) with a working voltage and emission current of 10 kV and 10 μA, respectively. Energy-dispersive X-ray spectroscopy (EDS) was performed under the working voltage of 15 kV for analyzing the elemental composition of the product. The microstructure was tested by using transmission electron microscopy (TEM, FEI Tecnai G2 F20, 200 kV, Boston, MA, USA). The pore size distributions and specific surface areas of the as-prepared products were evaluated via the Brunauer–Emmett–Teller (BET, GEMINI VII 2390, Norcross, GA, USA) method at 77 K. The pore size distribution was studied via the Barrett–Joyner–Halenda approach using desorption isotherms.

### 2.3. Electrochemical Performance Measurements

The CHI660E electrochemical workstation was employed to assess the electrochemical performance of the as-prepared samples. Polytetrafluoroethylene, acetylene black, and Gr/MoS_2_ heterostructures (8 mg) at a weight ratio of 5:15:80 were deposited on a piece of Ni foam (1 cm × 1 cm), and the Ni foam was pressed down by applying a hydraulic pressure of 8.0 MPa (working electrode). Pt and Hg/HgO were employed as the counter and reference electrodes, respectively. In addition, a 3.0-M KOH solution was used as the electrolyte. Galvanostatic charge–discharge (GCD), cyclic voltammetry (CV), and long-term cycle stability tests were performed using a three-electrode measurement technique. Electrochemical impedance spectroscopy (EIS) was performed using a PARSTAT2273 electrochemical workstation.

### 2.4. Fabrication of the Asymmetric Supercapacitive Device

The electrochemical properties of the MoS_2_/Gr heterostructures were examined using a two-electrode system. The working electrode was fabricated via an approach identical to the three-electrode technique. In addition, 12 mL of distilled water, 1.65 g of polyvinyl alcohol (PVA), and 2.15 g of KOH were mixed at 80 °C for 40 min to produce a gel electrolyte. The activated materials (MoS_2_/Gr heterostructures (8 mg) and activated carbon (8 mg)) were then wrapped with a gel electrolyte. After 5–7 min, a layer of solid electrolyte (PVA-KOH) was formed. Finally, the two-electrode devices were fabricated by pressing the two electrodes together using a sheet out roller.

## 3. Results

### 3.1. Crystal phase, Structure, and Microstructure Properties

**Figure 1:**
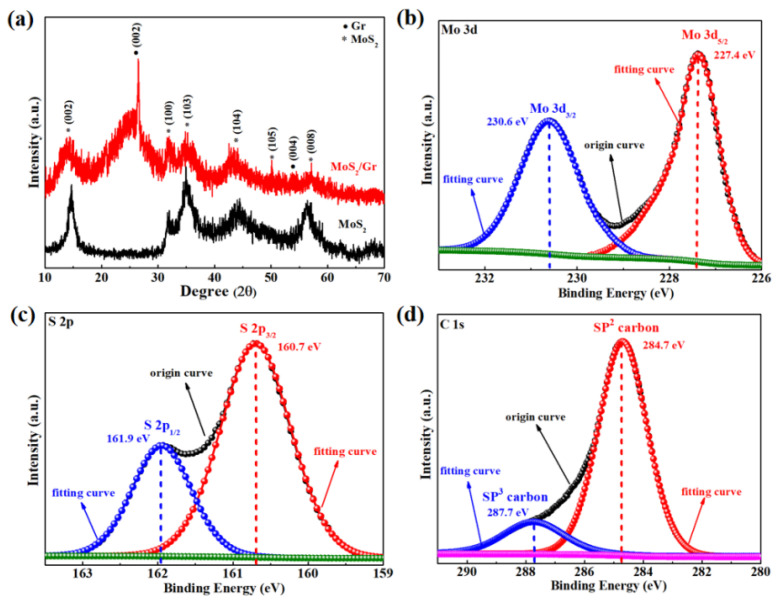
(**a**) XRD pattern of the pristine MoS_2_ and MoS_2_/Gr heterostructure. (**b**–**d**) High-resolution Mo 3d, S 2p, and C 1s XPS data, respectively.

The crystallinity and elemental composition of the surface of the fabricated samples were assessed using XRD and XPS, respectively. Figure 1a shows the XRD pattern of the pristine MoS_2_ and MoS_2_/Gr heterostructure. In this XRD pattern, the peaks at 26.58 and 54.56° can be attributed to the (002) and (004) planes of Gr, respectively (JCPDS No. 75-1621) [18,28], while those at 14.36°, 33.75°, 39.92°, 43.66°, 49.56°, and 58.96° correspond to the (002), (100), (103), (104), (105), and (008) crystallographic planes of MoS_2_, respectively (JCPDS No. 77-1716) [29]. In comparison, after the combination of MoS_2_ with Gr, the intensity of MoS_2_ in MoS_2_/Gr was reduced, confirming that the MoS_2_/Gr heterostructure was successfully synthesized. The full survey XPS spectrum is displayed in Figure S1 in the Supporting Information, which reveals that the Mo, S, and C elements are present in the as-prepared sample. The high-resolution XPS spectra of the MoS_2_/Gr heterostructure are shown in Figure 1b–d. The Mo 3d XPS spectrum (Figure 1b) shows two prominent peaks at binding energies of 227.4 eV (Mo 3d_5/2_) and 230.6 eV (Mo 3d_3/2_), which can be assigned to the Mo^4+^ in MoS_2_ [30]. The S 2p XPS spectrum (Figure 1c) exhibits two peaks at 161.9 eV (S 2p_1/2_) and 160.7 eV (S 2p_3/2_) corresponding to the S^2−^ in MoS_2_ [31]. In the C 1s XPS spectrum, the two main peaks present at 284.7 and 287.7 eV can be attributed to the sp^2^ and sp^3^ hybridized carbon in Gr, respectively [32].

**Figure 2:**
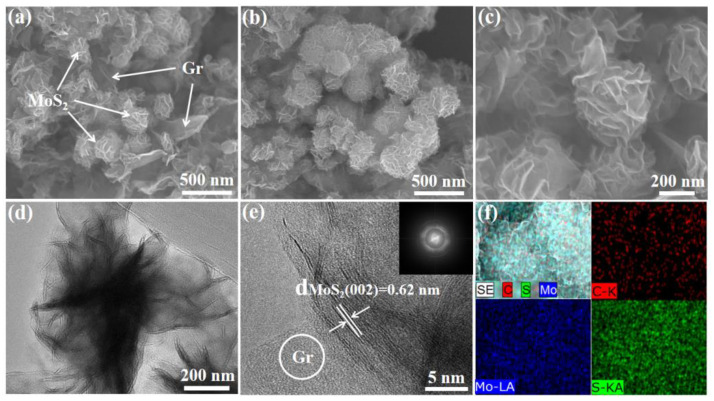
(**a**,**b**) Low-resolution and (**c**) high-resolution SEM images of the MoS_2_/Gr heterostructure. (**d**) TEM image of the MoS_2_/Gr heterostructure. (**e**) HRTEM image of the MoS_2_/Gr heterostructure (inset, the corresponding SAED image). (**f**) EDX mapping of C, Mo, and S.

Furthermore, SEM and TEM were employed to assess the surface morphology and microstructure of the as-prepared samples. Figure S2 (Supporting Information) presents the SEM image of pristine MoS_2_, suggesting that flower-shaped structures were formed. The low-resolution SEM images in Figure 2a,b indicate that flower-shaped MoS_2_ was successfully grown on the Gr layer. It is worth noting that the growth of flower-like MoS_2_ on the Gr layer improves the surface area of the MoS_2_/Gr heterostructure, providing it with abundant surface active sites, enhancing the structural stability of the electrode and further significantly improving the electrochemical performance of the material [33,34,35]. The high-resolution SEM image in Figure 2c shows that the flower-like MoS_2_ is made up of a large number of MoS_2_ nanoflakes, of 20 nm in thickness. Such thin MoS_2_ nanoflakes not only facilitate ion transfer between the electrolyte and electrodes, but also boost electrical conductivity. The TEM image in Figure 2d reveals that the Gr layer is decorated with flower-shaped MoS_2_, which is in good agreement with the SEM images shown in Figure 2a,b. The high-resolution TEM (HRTEM) and corresponding selected area electron diffraction (SAED) image is shown in Figure 2e and the inset of Figure 2e. As shown, there is a lattice spacing of the material of around 0.62 nm that can be assigned to the (002) crystallographic planes of the hexagonal lattice of MoS_2_. The lattices of MoS_2_ and Gr can be seen to be well-interfaced, indicating that the MoS_2_/Gr heterostructure was successfully fabricated. It should be noted that such a heterostructure significantly promotes the structural stability of the electrode, further leading to an improvement in its electrochemical cycling stability. Figure 2f and Figure S3 (Supporting Information) show the energy dispersive X-ray (EDX) mapping and spectrum of the MoS_2_/Gr heterostructure, in which the uniform distribution of the Mo, C, and S elements corroborates the formation of the material.

### 3.2. Electrochemical Performance of the MoS_2_/Gr Heterostructure

**Figure 3:**
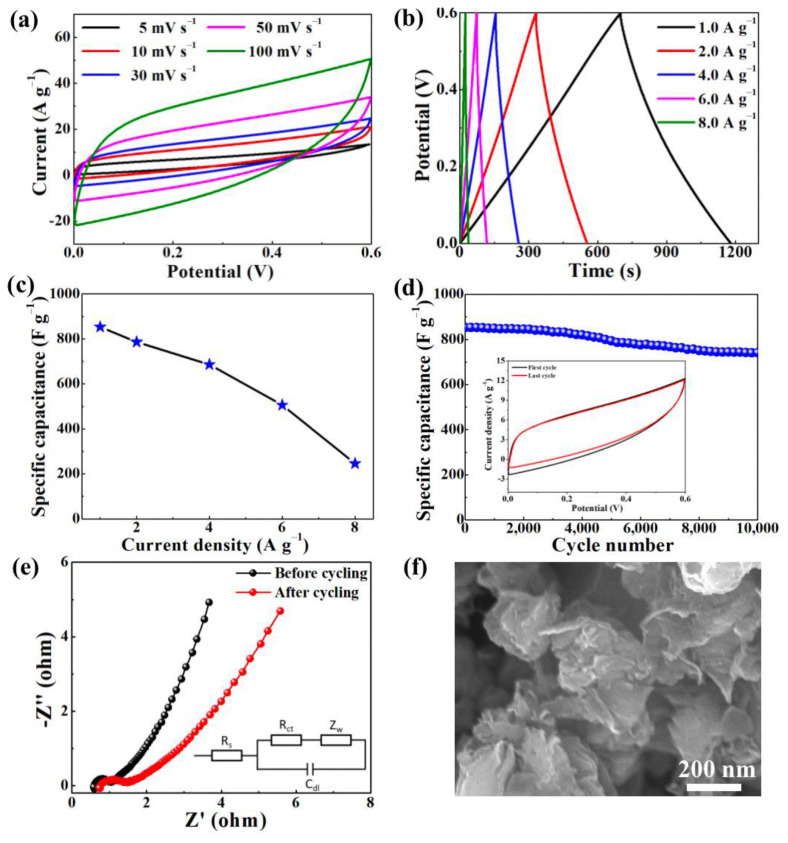
(**a**) CV curves of the MoS_2_/Gr electrode, recorded at different scan rates. (**b**) GCD curves of the MoS_2_/Gr electrode at different current densities. (**c**) Specific capacitance of the MoS_2_/Gr electrode at different current densities. (**d**) Cycling performance of the MoS_2_/Gr electrode at a scan rate of 30 mV s^−1^ (inset, the CV curves before and after cycling test). (**e**) EIS data of the MoS_2_/Gr electrode before and after 10,000 cycles (inset, the equivalent circuit of the MoS_2_/Gr electrode). (**f**) SEM image of the MoS_2_/Gr electrode after 10,000 cycles.

Figure 3a shows the CV curves of the MoS_2_/Gr electrode recorded at different scan rates in the range of 5–100 mV s^−1^, from which it can be seen that the current density gradually increases with an increase in the scan rate. The CV curve remained the same shape, even at a high scan rate of 100 mV s^−1^, indicating the remarkable capacitive behavior of the MoS_2_/Gr heterostructure [22,36]. The GCD curves (Figure 3b) of the MoS_2_/Gr electrode were recorded at various current densities in the range of 1–8 A g^−1^. Figure 3b shows the electrochemical capacitance of the MoS_2_/Gr electrode at various current densities (the equation used to calculate the electrochemical capacitance is provided in the Supporting Information). The electrochemical capacitance of the MoS_2_/Gr electrode was found to reach 853 F g^−1^ at 1 A g^−1^, as shown in Figure 3c. From the electrochemical measurements, it can be seen that as the current density was increased to 8 A g^−1^ and the electrochemical capacitance reached 225 F g^−1^. This phenomenon is due to the MoS_2_/Gr electrode having high electrical conductivity and a large surface area, as well as the synergistic effect of the Gr layer and flower-like MoS_2_ resulting in the fast diffusion and transfer of ions in the electrolyte. Moreover, the electrochemical performance of the MoS_2_-based electrode is shown in Figure S4 (Supporting Information); it shows an electrochemical capacitance of 227 F g^−1^ at 1 A g^−1^. In comparison, the MoS_2_/Gr electrode presents a higher electrochemical capacitance than that of MoS_2_, indicating that Gr can greatly enhance the electrochemical capacitance of MoS_2_. As shown in Figure S4a (Supporting Information) and Figure 3a, the CV curves of MoS_2_ and MoS_2_/Gr present a typical electrical double layer property. In general, for electrical double layer SCs, the electrochemical current is caused by the adsorption/desorption of the ions on the surface of the electrode. Therefore, for the electrode with a large surface area, more ions will be accumulated, and the reaction kinetic can be further optimized, thereby resulting in high electrochemical performance. To evaluate the surface area of the MoS_2_/Gr heterostructure, nitrogen (N_2_) adsorption-desorption isothermal analysis was performed at 77 K. The BET analysis (Figure S5 in the Supporting Information) indicates that the MoS_2_/Gr heterostructure has a large specific surface area of 95.2 m^2^/g, which significantly enhances its electrochemical performance [37,38]. Furthermore, compared with studies reported in the literature [39,40,41,42,43,44,45,46,47,48] (please see Table 1), the electrochemical capacitance of the MoS_2_/Gr heterostructure in this work is much higher, indicating that it is an excellent candidate for use in high-performance SCs. To investigate the electrical conductivity of the MoS_2_/Gr electrode, EIS measurements were performed before and after cycling stability tests were carried out over a frequency range from 0.01 Hz to 100 kHz with an alternating current voltage amplitude of 5 mV. The EIS curves and the corresponding equivalent circuit of the MoS_2_/Gr electrode before and after the 10,000 cycles test are shown in Figure 3e and the inset of Figure 3e. Compared with that of before cycling, the MoS_2_/Gr electrode exhibited a slight change in the solution resistance (R_s_) and charge-transfer resistance (R_ct_) after 10,000 cycles, indicating its excellent electrical conductivity [49]. Figure 3f shows an SEM image of the MoS_2_/Gr heterostructure after long-term cycling testing. A comparison of the SEM images shown in Figure 2b,c reveals that there are no significant changes in the structure of the MoS_2_/Gr heterostructure observed compared to before the cycling performance testing, suggesting that the MoS_2_/Gr heterostructure exhibits excellent structural stability.

**Figure 4:**
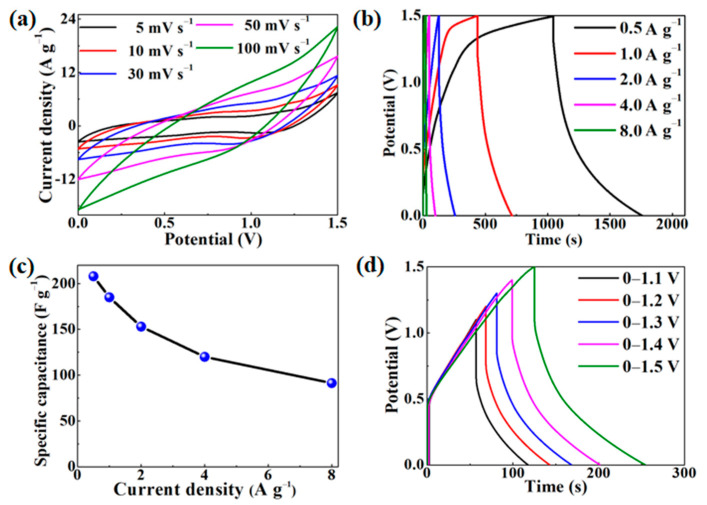

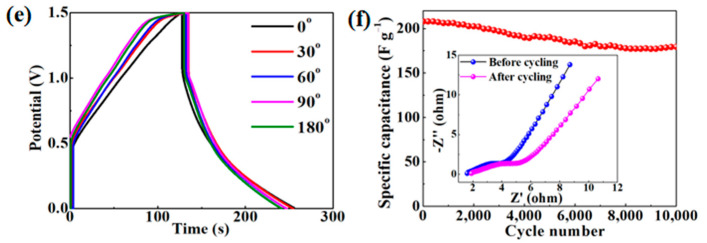
(**a**) CV curves of the flexible asymmetric device, recorded at different scan rates. (**b**) GCD curves of the flexible asymmetric device recorded at different current densities. (**c**) Specific capacitance of the flexible asymmetric device recorded at different current densities. (**d**) GCD curves of the flexible device recorded at a current density of 5 A g^−1^ over different potential ranges (0–1.1 V to 0–1.5 V). (**e**) GCD curves of the flexible device with different bending angles at a current density of 5 A g^−1^. (**f**) Cycling performance of the flexible asymmetric device at a current density of 1 A g^−1^ and EIS data of the flexible ASC device before and after 10,000 cycles (inset).

To assess the electrochemical capacitance performance of the MoS_2_/Gr heterostructure electrode for use in practical applications, a flexible ASC device was fabricated using the MoS_2_/Gr heterostructure and activated carbon as positive and negative electrodes, respectively. Figure S6 (Supporting Information) shows the CV curves of the positive and negative electrode materials recorded at a scan rate of 30 mV s^−1^. The CV curves (Figure 4a) of the flexible device were obtained at scan rates in the range of 5–100 mV s^−1^ over a large voltage window from 0 to 1.5 V. The CV curves exhibit no obvious distortion upon an increase in the scan rate, showing the superior charge–discharge reversibility of the device. GCD curves of the ASC device were recorded at different current densities (Figure 4b), with calculated electrochemical capacitances of 208, 185, 153, 120, and 92 F g^−1^ at current densities of 0.5, 1.0, 2.0, 4.0, and 8.0 A g^−1^, respectively (Figure 4c). The electrochemical capacitance was observed to remain at approximately 44.2% of the initial value at a current density of 8.0 A g^−1^, indicating that the ASC device exhibits excellent electrochemical properties. Moreover, GCD curves over different voltage windows (0–1.1 V to 0–1.5 V) were measured at 2 A g^−1^, with the results shown in Figure 4d. The GCD curves retained their shapes, even at potentials of as high as 1.5 V, revealing the ideal capacitance features of the electrode [50].

### 3.3. Stability and Flexibility Properties

It is well known that electrochemical durability is a crucial parameter of SCs. Hence, the electrochemical cycling stability of the MoS_2_/Gr electrode was also assessed at 1 A g^−1^ over 10,000 cycles, with the results shown in Figure 3d. The CV curves of before and after the 10,000 cycles test are shown in the inset of Figure 3d. It was found that 89.6% of the initial electrochemical capacitance of the MoS_2_/Gr electrode was retained after long-term cycling, indicating that the MoS_2_/Gr heterostructure exhibits excellent electrochemical cycling stability. This superior electrochemical cycling stability can be attributed to the growth of flower-shaped MoS_2_ on the Gr, which significantly enhances the structural stability of the electrode.

Furthermore, the ASC device was tested at various angles (0°, 30°, 60°, 90°, and 180°) to examine its flexibility. Figure 4e shows the GCD curves of the flexible ASC device twisted at different bending angles at 2 A g^−1^. The corresponding CV curves are presented in Figure S7 in the Supporting Information. Note that the shapes of the GCD and CV curves are almost identical after multiple bending tests, indicating the excellent flexibility of the device. Figure 4f presents the long-term cycling stability of the flexible ASC device at 0.5 A g^−1^, in which it can be seen that 86.5% of the electrochemical capacitance was retained after 10,000 cycles, indicating that the ASC device exhibits outstanding cycling durability. The EIS results obtained before and after cycling durability testing are shown in the inset of Figure 4f. The charge-transfer resistance (R_ct_) of the ASC device exhibits no appreciable changes after cycling durability testing, confirming that it exhibits good electrical conductivity.

### 3.4. Ragone Plot Properties

**Figure 5:**
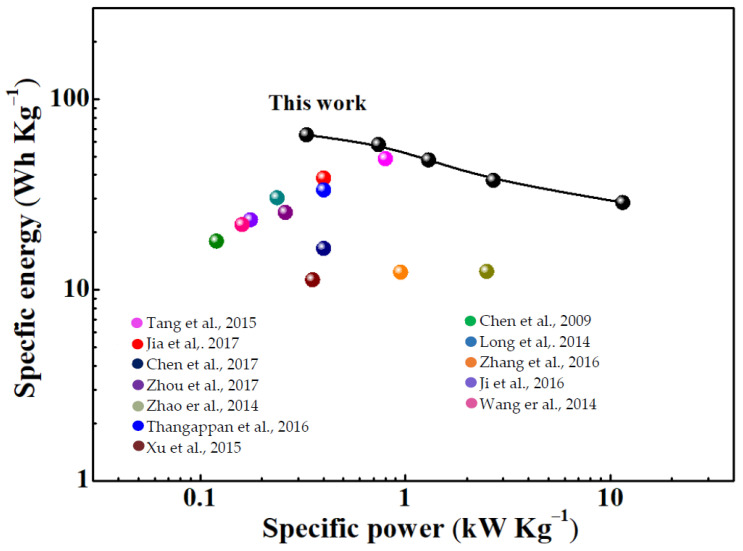
Ragone plots (power density vs. energy density) of the flexible ASC device [6,25,47,50,51,52,53,54,55,56,57,58].

The energy and power densities (Figure 5) of the ASC device were estimated from Figure 4b. A high specific energy (65 Wh kg^−1^) was achieved at a specific power of 0.33 kW kg^−1^, and the specific energy reached 28.7 W h kg^−1^ at a specific power of 11.5 kW kg^−1^. These values are much higher than those of the results in the literature (Figure 5) [6,25,47,50,51,52,53,54,55,56,57,58], suggesting that the MoS_2_/Gr heterostructure composite is an outstanding candidate for use in eco-friendly, sustainable, and high-performance SCs.

## 4. Conclusions

A MoS_2_/Gr heterostructure was successfully fabricated via a cost effective and simple strategy. The SEM and TEM images indicate the successful growth of flower-like MoS_2_ on the Gr layer, which increases the surface area, enhances the structural stability, and boosts the electrical conductivity of the electrode. In three-electrode measurements performed on the MoS_2_/Gr heterostructure, the electrochemical capacitance reached 853 F g^−1^ at 1.0 A g^−1^. Furthermore, the flexible ASC device composed of the MoS_2_/Gr heterostructure and activated carbon exhibited a high electrochemical specific capacitance of 208 F g^−1^ at 0.5 A g^−1^, in addition to a high specific energy of 65 Wh kg^−1^ at a specific power of 0.33 kW kg^−1^. More importantly, after 10,000 cycles, 86.5% of the electrochemical capacitance was retained, indicating the long-term cycling durability of the device. These excellent electrochemical properties indicate that the MoS_2_/Gr heterostructure composite is a promising candidate for use in flexible, sustainable, and advanced SCs.

## Figures and Tables

**Table 1:** Comparison of the specific capacitance of the electrode in this work compared with those based on MoS_2_/Gr hybrids reported in the literature.

Materials	Specific Capacitance/F g^−1^	Current Density/ Scan Rate	Potential Range	Electrolyte	Reference
MoS_2_/Gr	853	1 A g^−1^	0~0.6 V	3 M KOH	This work
MoS_2_/3D Gr	410	1 A g^−1^	−0.9~−0.2 V	1M Na_2_SO_4_	Ref. [39]
MoS_2_-O@MoS_2_	205	5 mV s^−1^	0~0.9 V	1M Na_2_SO_4_	Ref. [40]
MoS_2_/3D Gr	169.37	1 A g^−1^	−0.9~0.1 V	0.1 M KOH	Ref. [41]
MoS_2_/carbon	676.4	1 A g^−1^	0~0.55 V	3 M KOH	Ref. [42]
MoS_2_/carbon spheres	411	1 A g^−1^	−0.9~−0.1 V	1 M Na_2_SO_4_	Ref. [43]
MoS_2_/rGO	218.3	1 A g^−1^	−1.0~−0.1 V	2 M KOH	Ref. [44]
MoS_2_ nanosheets	119.38	5 mV s^−1^	−1.0~0 V	1 M Li_2_SO_4_	Ref. [45]
MoS_2_ nanosheets	133	1 A g^−1^	−0.9~−0.1 V	1 M KCl	Ref. [46]
MoS_2_/polypyrrole	350	1 A g^−1^	−0.8~−0.2 V	0.5 M Na_2_SO_4_	Ref. [47]
MoS_2_/C	712.6	1 A g^−1^	0~0.4 V	6 M KOH	Ref. [48]

## Data Availability

All data needed to evaluate the conclusions in the paper are present in the paper and/or the Supplementary Materials. Additional data related to this paper may be requested from the authors.

## Supplementary Materials

The following supporting information can be downloaded at: https://www.mdpi.com/article/10.3390/mi14020297/s1. Figure S1. Full X-ray photoelectron spectrum of the Gr/MoS_2_ heterostructures; Figure S2. The SEM image of pure MoS_2_; Figure S3. The EDX spectrum results of the MoS_2_/Gr heterostructure; Figure S4. (a) CV curves of the MoS_2_ electrode at the different scan rates, (b) GCD curves of the MoS_2_ electrode at different current densities, (c) the specific capacitance of the MoS_2_ electrode at different current densities; Figure S5. (a) N_2_ adsorption–desorption isotherm curves and (b) pore-size distribution of the MoS_2_/Gr heterostructure; Figure S6. CV curves of the negative and positive electrodes, obtained at a scan rate of 30 mV s^−1^; Figure S7. CV curves of the flexible device with different bending angles, obtained at a scan rate of 30 mV s^−1^.
