# Supplementary material for: High-Performance Flexible Energy Storage Devices Based on Graphene Decorated with Flower-Shaped MoS2 Heterostructures
Source: Micromachines (Basel). 2023 Jan 23;14(2):297. doi: 10.3390/mi14020297 (PMC9967960; doi:10.3390/mi14020297)
Supplement: Supplementary file 1 [file micromachines-14-00297-s001.zip › micromachines-2163298-supplementary.pdf]

Supporting Information

High-performance flexible energy storage devices based on graphene decorated with flower shaped MoS_2 heterostructures

Yongteng Qian[1,2,*], Zhiyi Lyu[2], Qianwen Zhang[2], Tae Hyeong Lee[2], Tae Kyu Kang[2], Minkyun Sohn[2], Lin Shen[3], Dong-Hwan Kim[3,*], and Dae Joon Kang[2,*]

[1]College of Pharmacy, Jinhua Polytechnic, Jinhua, Zhejiang Province, 321007, P. R. China

[2]Department of Physics, Sungkyunkwan University, 2066, Seobu-ro, Jangan-gu, Suwon, Gyeonggi-do 16419, Republic of Korea

[3]School of Chemical Engineering, Sungkyunkwan University, 2066, Seobu-ro, Jangan-gu, Suwon, Gyeonggi-do 16419, Republic of Korea

*Authors to whom the correspondence should be addressed: qytwork@skku.edu; djkang@skku.edu; dhkim1@skku.edu

Calculations

The specific capacitance was estimated from the GCD results using the following equation:

$$Cs = (\Delta t \times I)/(\Delta V \times m)$$

The energy density (E) can be determined using the following equation:

$$E = 0.5C_S \times (\Delta V)^2$$

The power density (P) can be determined using the following equation:

$$P = E/\Delta t$$

where Cs (F g^{-1}) represents the specific capacitance of the electrode; Δt (s) represents the discharge time; I (A) represents the discharge current; m (g) represents the mass of the activated materials; and ΔV (V) represents the discharge potential window.

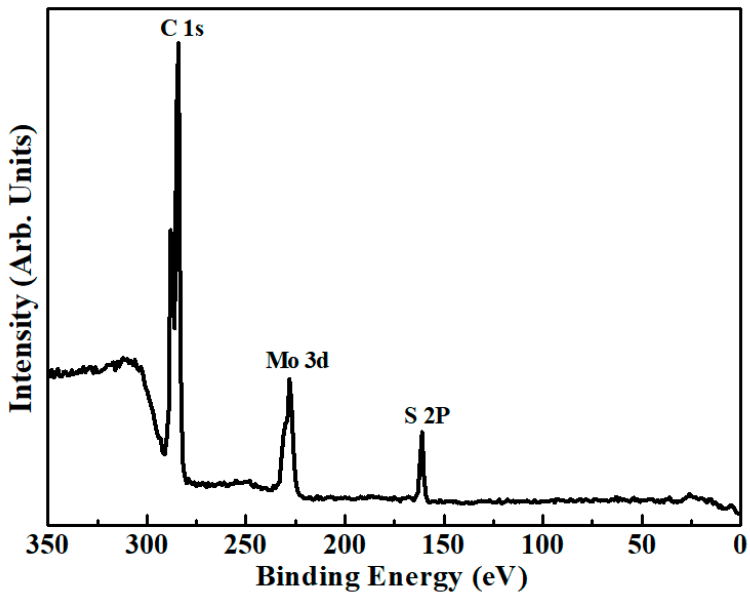

Figure S1. Survey XPS spectrum of the MoS_2/Gr heterostructure.

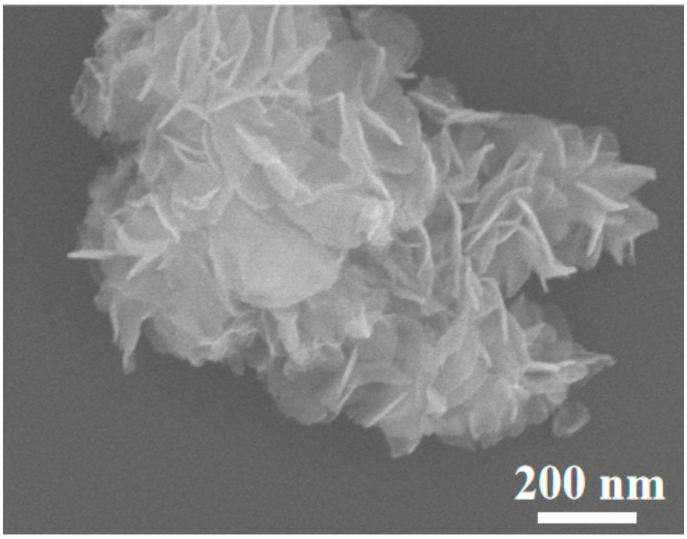

Figure S2. The SEM image of pure MoS_2.

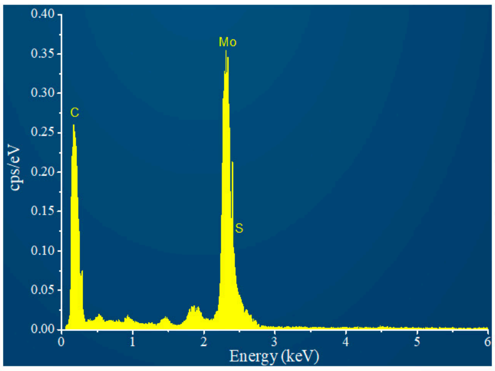

El	AN	Series	unn. C [wt.%]	norm. C [wt.%]	Atom. C [at.%]	Error (1 Sigma) [wt.%]
Mo	42	L-series	45.78	47.64	16.23	1.74
S	16	K-series	29.45	30.65	34.32	1.14
C	6	K-series	20.86	21.71	49.45	6.67
		Total:	96.09	100.00	100.00	

Figure S3. The EDX spectrum results of the MoS_2/Gr heterostructure.

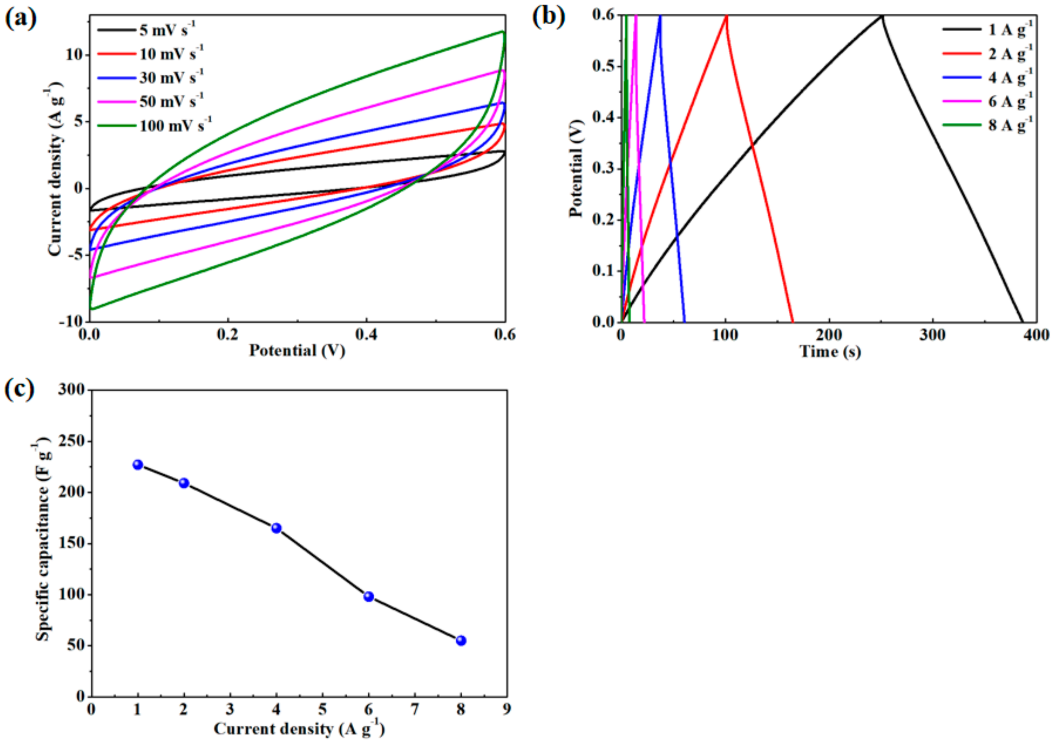

Figure S4. (a) CV curves of the MoS_2 electrode at the different scan rates, (b) GCD curves of the MoS_2 electrode at different current densities, (c) the specific capacitance of the MoS_2 electrode at different current densities.

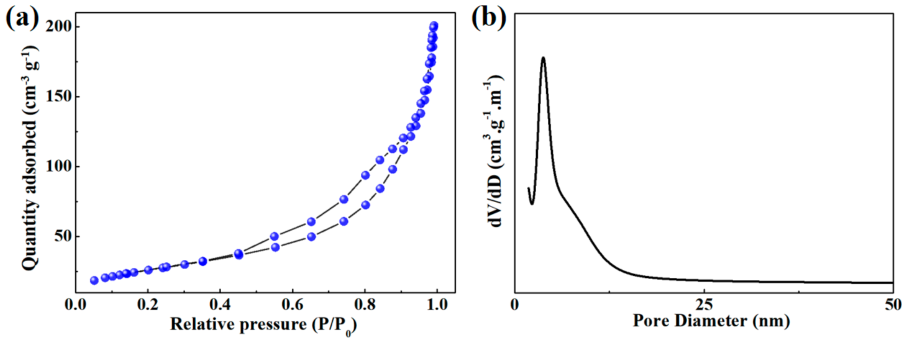

Figure S5. (a) N_2 adsorption–desorption isotherm curves and (b) pore-size distribution of the MoS_2/Gr heterostructure.

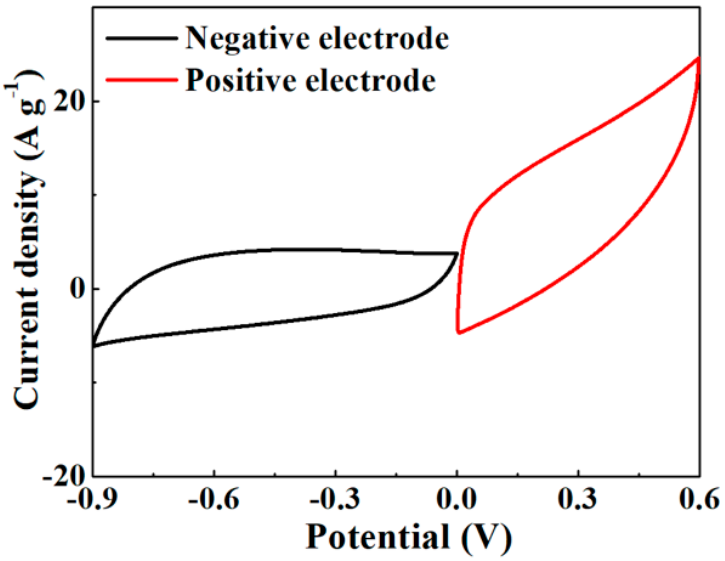

Figure S6. CV curves of the negative and positive electrodes, obtained at a scan rate of 30 mV s^{-1}.

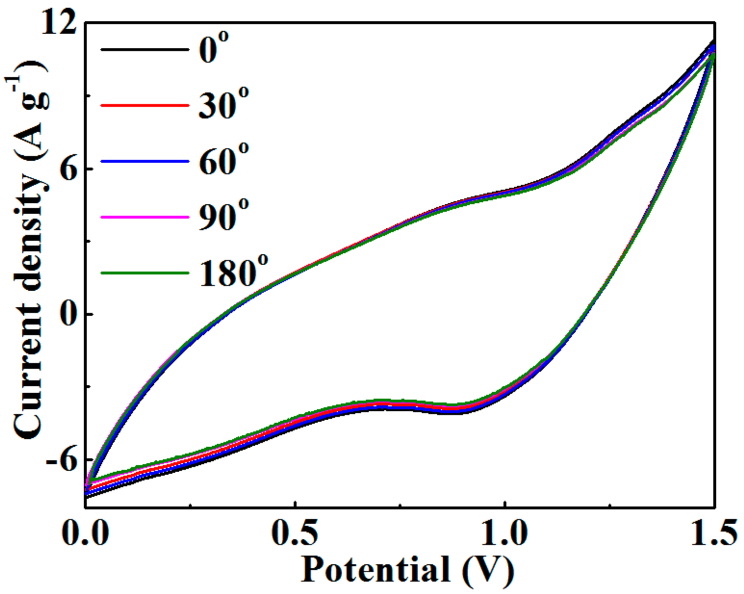

Figure S7. CV curves of the flexible device with different bending angles, obtained at a scan rate of 30 mV s^{-1}.
